# Azithromycin and Chloramphenicol Diminish Neutrophil Extracellular Traps (NETs) Release

**DOI:** 10.3390/ijms18122666

**Published:** 2017-12-08

**Authors:** Weronika Bystrzycka, Aneta Manda-Handzlik, Sandra Sieczkowska, Aneta Moskalik, Urszula Demkow, Olga Ciepiela

**Affiliations:** 1 Department of Laboratory Diagnostics and Clinical Immunology of Developmental Age, Medical University of Warsaw, 02-091 Warsaw, Poland ; weronika.bystrzycka@wum.edu.pl (W.B.); urszula.demkow@litewska.edu.pl (U.D.); 2 Student’s Scientific Group at Department of Laboratory Diagnostics and Clinical Immunology of Developmental Age, Medical University of Warsaw, 02-091 Warsaw, Poland; sandra.sieczkowska@gmail.com (S.S.); aneta.moskalik@gmail.com (A.M.); 3 Postgraduate School of Molecular Medicine, Medical University of Warsaw, 02-091 Warsaw, Poland

**Keywords:** azithromycin, chloramphenicol, neutrophil extracellular traps

## Abstract

Neutrophils are one of the first cells to arrive at the site of infection, where they apply several strategies to kill pathogens: degranulation, respiratory burst, phagocytosis, and release of neutrophil extracellular traps (NETs). Antibiotics have an immunomodulating effect, and they can influence the properties of numerous immune cells, including neutrophils. The aim of this study was to investigate the effects of azithromycin and chloramphenicol on degranulation, apoptosis, respiratory burst, and the release of NETs by neutrophils. Neutrophils were isolated from healthy donors by density-gradient centrifugation method and incubated for 1 h with the studied antibiotics at different concentrations (0.5, 10 and 50 μg/mL—azithromycin and 10 and 50 μg/mL—chloramphenicol). Next, NET release was induced by a 3 h incubation with 100 nM phorbol 12-myristate 13-acetate (PMA). Amount of extracellular DNA was quantified by fluorometry, and NETs were visualized by immunofluorescent microscopy. Degranulation, apoptosis and respiratory burst were assessed by flow cytometry. We found that pretreatment of neutrophils with azithromycin and chloramphenicol decreases the release of NETs. Moreover, azithromycin showed a concentration-dependent effect on respiratory burst in neutrophils. Chloramphenicol did not affect degranulation, apoptosis nor respiratory burst. It can be concluded that antibiotics modulate the ability of neutrophils to release NETs influencing human innate immunity.

## 1. Introduction

Neutrophils are the most abundant immune cells in human peripheral blood, and play a crucial role in the innate immune response by defending the body against pathogens. They are major antimicrobial effector cells designed to kill microbes by several strategies: degranulation, phagocytosis, generation of reactive oxygen species (ROS), and by the—recently discovered—release of neutrophil extracellular traps (NETs) in a process called NETosis. NETs are fragile fibers of decondensed chromatin decorated with antimicrobial proteins and histones [1]. These web-like structures are released from the cell to form a physical barrier for the pathogens that limits their spread throughout the organism, and to generate a high local concentration of antimicrobial factors. Despite their beneficial role, NETs have been also reported to contribute to the development of several diseases, including rheumatoid arthritis, diabetes, and thrombosis [2]. Therefore, a substantial effort is being made to identify agents modulating the function of neutrophils, as this may help develop new therapeutic strategies for the treatment of patients suffering from NET-related diseases.

It is proposed that antibiotics may act as agents affecting NET release. The influence of antibiotics on the immune system has been an object of investigation for nearly 70 years [3]. There are many reports regarding their immunomodulating effect on granulocytic and lymphocytic functions [4,5,6]. Hoeben et al. have demonstrated that antibiotics affect the respiratory burst activity and phagocytosis of neutrophils [7]. Moreover, Lai et al. report that azithromycin-loaded neutrophils are more effective in bacterial killing by phagocytosis [8]. One of the first groups to describe the impact of antibiotics on the release of NETs was Jerjomiceva et al., who observed enhanced NETosis in bovine granulocytes pretreated with enrofloxacin [9]. To date, there have been few studies regarding the impact of antibiotics on NET release; thus, we decided to widen knowledge about this topic. There are findings concerning the link between antibiotic treatment and NETosis; however, these predominantly describe the induction of NET release by the addition of bacteria to the cell containing the antibiotic [10,11]. The aim of this study was to investigate changes in the functions of neutrophils (NETosis, degranulation, oxidative burst) caused by the presence of antibiotics in the cell medium.

## 2. Results

### 2.1. Degranulation

**Figure 1:**
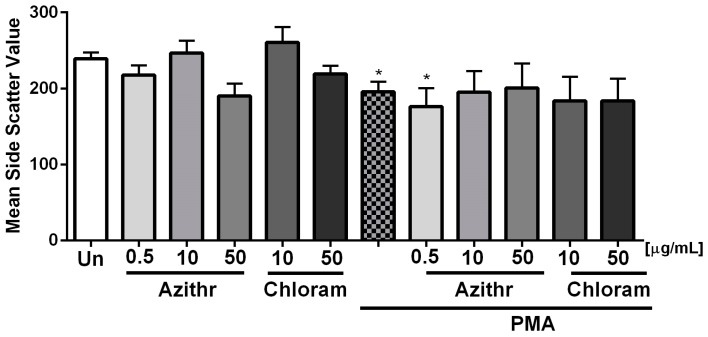
Effect of azithromycin and chloramphenicol on neutrophil degranulation assessed by measuring the granularity degree of neutrophils in the side scatter channel (SSC). 100 nM phorbol 12-myristate 13-acetate (PMA) was used as a positive control of degranulation (*n* ≥ 3) (* *p* ≤ 0.05 vs. unstimulated (Un) cells).

Phorbol 12-myristate 13-acetate (PMA) was used as an inducer of degranulation during analysis of the morphological complexity of neutrophils in the side scatter channel (SSC) by flow cytometry. We found that none of the studied antibiotics alone caused cell degranulation (Figure 1). Instead, we found that azithromycin at doses of 10 and 50 μg/mL prevented cell degranulation upon PMA treatment.

### 2.2. Apoptosis

**Figure 2:**
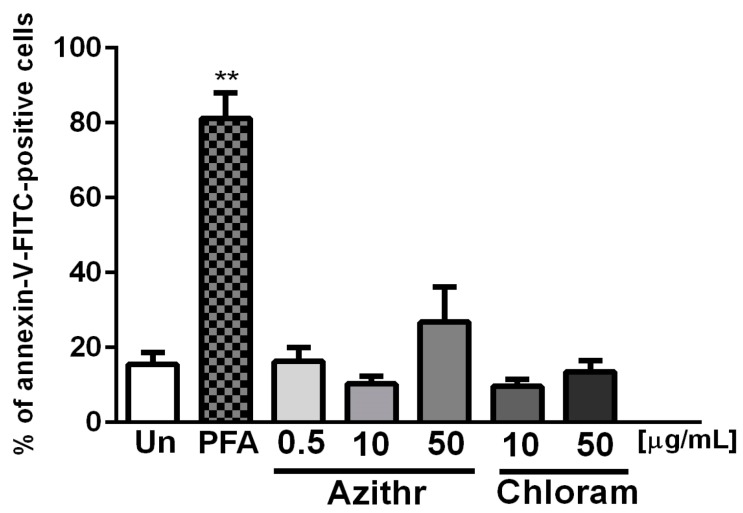
Effect of azithromycin and chloramphenicol on apoptosis assessed by annexin V-FITC and propodium iodide binding; paraformaldehyde (PFA) constituted positive control (*n* = 6) (** *p* ≤ 0.01 vs. unstimulated cells (Un)).

Incubation of granulocytes with antibiotics did not induce cells apoptosis, although a slight, non-significant increase in the number of annexin V positive neutrophils compared to control, untreated cells was observed after the incubation of the cells with 50 μg/mL azithromycin (Figure 2).

### 2.3. Oxidative Burst

**Figure 3:**
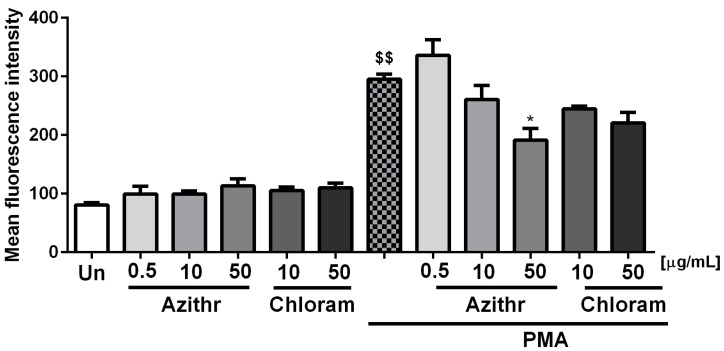
Effect of azithromycin and chloramphenicol on respiratory burst (*n* = 6), $$ *p* ≤ 0.01 vs. unstimulated cells (Un), * *p* ≤ 0.05 vs. phorbol 12-myristate 13-acetate (PMA).

None of the studied antibiotics alone affected oxidative burst in neutrophils. However, pretreatment of cells with 50 μg/mL azithromycin significantly inhibited ROS production after stimulation with PMA (*p* ≤ 0.05) (Figure 3).

### 2.4. NETosis

**Figure 4:**
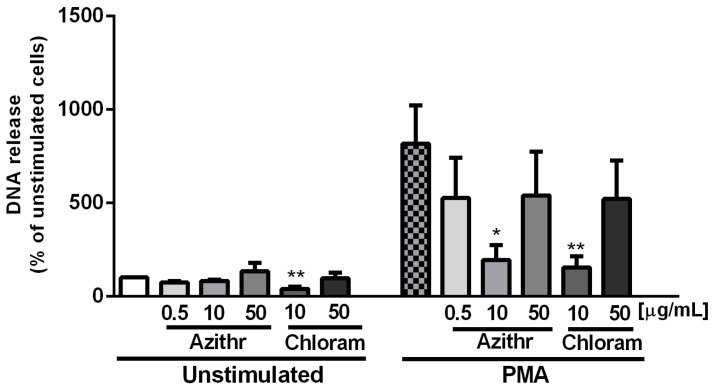
Effect of azithromycin and chloramphenicol on NET release. 100 nM phorbol 12-myristate 13-acetate (PMA) was added to stimulate the release of NETs, neutrophils treated with antibiotics without stimulation served as the negative control (*n* = 6).

**Figure 5:**
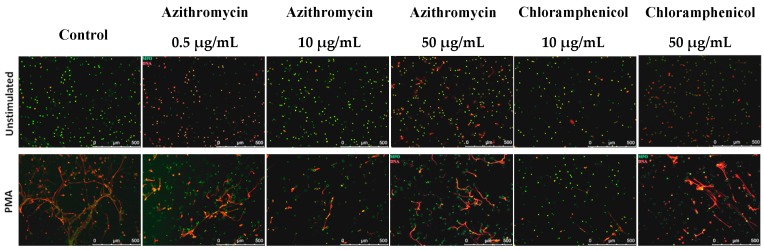
Visualization of the release of NETs performed by fluorescence microscopy after incubation with azithromycin and chloramphenicol, with or without stimulation with 100 nM PMA. The green color represents MPO, red DNA (*n* = 6).

Our studies revealed that pretreatment of the cells with 10 μg/mL chloramphenicol alone led to reduced spontaneous release of NETs by cells (*p* ≤ 0.01). Moreover, we found that incubation of the cells with 10 μg/mL azithromycin and 10 μg/mL chloramphenicol caused a significant decrease in NET release after stimulation with PMA (*p* ≤ 0.05 for 10 μg/mL azithromycin and *p* ≤ 0.01 for 10 μg/mL chloramphenicol (Figure 4). Fluorescent microscopy confirmed the above results (Figure 5).

## 3. Discussion

In this study, we were able to show that azithromycin and chloramphenicol can modify the functions of neutrophils. Although these antibiotics at the tested concentrations do not affect neutrophil degranulation and apoptosis, they may inhibit NETosis and affect production of ROS.

Azithromycin belongs to the group of macrolides, which are able to accumulate in tissues, most importantly in white blood cells [12]. Therefore, intracellular activity of macrolides against pathogens is much stronger than that of other antibiotics [13], as they can be easily transported to the site of infection [14]. In our study, azithromycin exerted a concentration-dependent effect on ROS production, and degranulation by PMA-stimulated neutrophils. Although this effect was significant only at concentrations of 50 μg/mL for ROS release, we observed that increasing concentrations of azithromycin tended to gradually decrease the ability of neutrophils to produce ROS. Regarding degranulation, azithromycin at high concentrations (10 and 50 μg/mL) did cause less effective degranulation of neutrophils after stimulation with PMA. Overall, this could suggest an inhibiting or stabilizing effect of azithromycin on stimulated neutrophils. What is more, azithromycin had an inhibitory effect on NET release, with a significant decrease at a concentration of 10 μg/mL azithromycin vs. PMA only. Surprisingly, this effect was no longer observed at a concentration of 50 μg/mL. As PMA is a ROS-dependent inducer of NETosis [15], and azithromycin showed an inhibitory effect on ROS production, we hypothesize that azithromycin at least partially influences the release of NETs by affecting ROS production. The fact that the highest concentration of azithromycin does not significantly inhibit NETosis despite a strong decrease in ROS production may be surprising. However, one should be aware that NET release is, without a doubt, a complex process, involving a number of molecular events, not only ROS production. Therefore, it cannot be excluded that at the highest concentration of azithromycin, other processes that promote NETosis may be upregulated.

Chloramphenicol was introduced for use in 1949; but after only 48 years, it was banned from use, as it had been reported to cause, among other things, aplastic anaemia. Nowadays, it is mostly used as a drug of last resort, but is still a first line drug for bacterial conjuctivitis [16]. To our knowledge, the impact of chloramphenicol on the process of NETosis has not been investigated, yet. In our study, chloramphenicol at a concentration of 10 μg/mL decreased the release of NETs after incubation with PMA. Moreover, pretreatment of cells with 10 μg/mL chloramphenicol reduced the number of cells undergoing spontaneous NETosis in unstimulated samples. Hoeben et al. reported that chloramphenicol may reduce myeloperoxidase (MPO) activity in bovine granulocytes [7]. Considering the fact that, during NETosis, chromatin decondensation occurs due to the synergistic work of MPO and neutrophil elastase [17], it seems feasible that chloramphenicol inhibits NETosis via the inhibition of MPO.

Antibiotics, aside from their antimicrobial properties, can induce an immunomodulating effect on cells [18,19,20,21], which varies depending on the antibiotic used, its concentration, and the target cells. In our previous studies, we have already demonstrated the impact of clindamycin, amoxicillin, gentamicine and cefotaxime on the release of NETs [22,23]. We found that amoxicillin induces NET release, and that gentamicine inhibits NETosis; meanwhile, we found that cefotaxime and clindamycin have no effect of NET release. Based on our current investigation, it can be concluded that both azithromycin and chloramphenicol may influence innate immunity by reducing the ability of neutrophils to release NETs. Overall, as the effects of different antibiotics on neutrophils are quite distinct, it cannot be excluded that the effect of a given drug may depend on its chemical composition and mechanism of action.

When interpreting data of our study, one should bear in mind the limitation that we analyzed the influence of antibiotics solely on the basis of NET formation induced with an artificial stimulus, PMA. Even though PMA is considered to mimic signals derived from microbes or immune products [24], the availability of data using physiological stimuli, e.g., lipopolysaccharide, would further support our conclusions, and it would be beneficial to perform such studies in the future. What is more, we would like to point out that the influence of antibiotics on neutrophils in vivo may be influenced by extracellular milieu, such as the changes in local pH during infection or availability of plasma proteins [25,26], whilst our studies were performed in protein-free medium, and the pH was stabilized with HEPES buffer.

NETs play an important role in the innate immune system, as they form a physical barrier for the pathogens that limits their spread throughout the organism, and generate a high local concentration of antimicrobial proteins, increasing their efficacy [1]. Disorders in NET formation have been shown to cause increased susceptibility to opportunistic infections. Despite their beneficial role, NETs have also been reported to damage cells located near the web-like structures, and contribute to the development of several diseases. As NETs constitute a considerable source of autoantigens, most often they are described as contributors to autoimmune diseases such as systemic lupus erythematosus and rheumatoid arthritis [2]. Identification of agents modulating the function of neutrophils has become an important goal for the scientific community, as it may help develop new therapeutic strategies for the treatment of patients suffering from NET-related diseases. When it comes to antimicrobial therapy, the choice of an antibiotic that decreases NETosis may impair natural mechanisms of innate immunity, while at the same time diminishing the release of autoantigens stimulating autoimmune processes. It would be thus advantageous for patients suffering from autoimmune diseases if doctors, whenever possible, chose antibiotics that negatively influenced NET release.

## 4. Materials and Methods

### 4.1. Antibiotics

Azithromycin and chloramphenicol were purchased from Sigma-Aldrich and diluted in protein-free RPMI medium supplemented with 10 mM HEPES (ThermoFisher Scientific, Waltham, MA, USA) to give a final concentration of 0.5, 10 and 50 μg/mL (azithromycin) or 10 and 50 μg/mL (chloramphenicol) in each experiment. The concentrations used in the study for chloramphenicol are the same as its therapeutic serum concentration, which is 2–50 μg/mL [27]. For azithromycin, we used concentrations similar to those achieved in white blood cells during therapy (0–40 μg/mL; serum concentrations: up to 0.4 μg/mL) [28].

### 4.2. Neutrophil Isolation and Preparation

Peripheral venous blood was collected from healthy donors into citrate tubes. All experiments were approved by Ethics Committee at Medical University of Warsaw. Written informed consent was obtained from each volunteer. Neutrophils were isolated by the density-gradient centrifugation method, as described previously [22].

In all the performed experiments, neutrophils were preincubated with the studied antibiotics for 1 h at 37 °C, 5% CO_2_.

### 4.3. Degranulation

Degranulation was analyzed using flow cytometry [9] (Cytomics FC500 Beckman Coulter, Beckman Coulter Inc., Brea, CA, USA) by assessing the granularity degree of neutrophils in the side scatter channel (SSC). For this purpose, 2.5 × 10^5^/mL neutrophils were incubated with the studied antibiotics for 1 h at 37 °C, 5% CO_2_. Positive control with 100 nM phorbol 12-myristate 13-acetate (PMA, Merck Millipore, Burlington, MA, USA) was used, whereas granulocytes with RPMI alone constituted negative control.

### 4.4. Apoptosis

Apoptosis was assessed with Annexin V Apoptosis Detection Kit FITC (eBioscience, Thermo Fisher Scientific, Waltham, MA, USA) by flow cytometry. After incubation with antibiotics for 1 h at 37 °C, 5% CO_2_, 2.5 × 10^5^/mL of granulocytes were washed, centrifuged, suspended in binding buffer and double-stained with annexin V-FITC and propidium iodide according to the manufacturer’s instructions. Positive control (apoptotic cells) was obtained by adding 4% final concentration (f.c.) paraformaldehyde (PFA) to the cell suspension. Negative control contained cells resuspended in RPMI.

### 4.5. Respiratory Burst

To analyze the respiratory burst in granulocytes, dihydrorhodamine 123 (DHR 123, (Thermo Fisher Scientific) was used as a fluorescent marker of the intracellular production of reactive oxygen species (ROS). For this purpose, 2.5 × 10^5^/mL of neutrophils were incubated with 4 μg/mL DHR123 for 30 min in 37 °C, 5% CO_2_. Subsequently, cells were incubated for 1 h at 37 °C, 5% CO_2_, with the studied antibiotics. 100 nM PMA, which is an efficient stimulator of ROS production, was used as a positive control. Fluorescence intensity of rhodamine 123 formed during oxygen burst was measured at the first fluorescence channel with flow cytometry.

### 4.6. NET Quantification

Isolated cells were seeded into 96-well black plates at density of 1 × 10^5^ cells/well (5 × 10^5^ cells/mL), treated with antibiotics or medium alone and left for 1 h at 37 °C, 5% CO_2_. Then, 100 nM PMA was added to stimulate NETs formation for 3 h at 37 °C, 5% CO_2_. Unstimulated granulocytes were used as control cells. Post stimulation, 500 mIU/mL of micrococcal nuclease (ThermoFisher Scientific) was added for 20 min at 37 °C to detach DNA from the bottom of the wells. Reaction was stopped with 5 mM EDTA, and then the plate was centrifuged at 415× *g* for 10 min. Then, supernatant was collected to black titer plates and 100 nM Sytox green fluorescent dye (Life Technologies, Waltham, MA, USA) was added to each well to measure the amount of extracellular DNA by fluorometry.

### 4.7. NET Visualization

NET formation was visualized using fluorescent microscopy. Briefly, neutrophils (2.5 × 10^4^ cells/well; 6.25 × 10^4^ cells/well) were seeded onto 8-well Lab-Tek Chamber Slides (ThermoFisher Scientific) and incubated for 1 h at 37 °C at 5% CO_2_ with antibiotics. Subsequently, 100 nM PMA was added to stimulate NETs release. After 3 h, samples were fixed with 4% f.c. PFA and washed 3 times with PBS. Cells were permeabilized with 0.1% Triton X (Sigma-Aldrich, St. Louis, MO, USA) and again washed with PBS. Samples were stained overnight with anti-myeloperoxidase-FITC monoclonal antibody (1:500, 4 °C, Abcam ab11729, Cambridge, UK). DNA was then counterstained with nucleic acid dye Sytox Orange (Life Technologies, Waltham, MA, USA). NETs were visualized with a Leica DMi8 microscope (Wetzlar, Germany).

### 4.8. Statistical Analysis

Statistical analysis was performed using GraphPad Prism v.6.0 (GraphPad Software, La Jolla, CA, USA). All values were analyzed with one-way ANOVA followed by a post-hoc test for paired data. All the results have been presented as mean ± standard error of the mean. Results were considered statistically significant at *p* < 0.05.

## Abbreviations

ROS	Reactive oxygen species
NETs	Neutrophil extracellular traps
MPO	Myeloperoxidase
PMA	Phorbol 12-myristate 13-acetate
PFA	Paraformaldehyde
SSC	Side scatter channel
f.c.	Final concentration

## References

[1] Manda A., Pruchniak M.P., Arazna M., Demkow U.A. (2014). Neutrophil extracellular traps in physiology and pathology. Cent.-Eur. J. Immunol..

[2] Branzk N., Papayannopoulos V. (2013). Molecular mechanisms regulating NETosis in infection and disease. Semin. Immunopathol..

[3] Munoz J., Geister R. (1950). Inhibition of phagocytosis by aureomycin. Proc. Soc. Exp. Biol. Med..

[4] Healy D.P., Silverman P.A., Neely A.N., Holder I.A., Babcock G.E. (2002). Effect of antibiotics on polymorphonuclear neutrophil apoptosis. Pharmacotherapy.

[5] Seklecki M.M., Quintiliani R., Maderazo E.G. (1978). Aminoglycoside antibiotics moderately impair granulocyte function. Antimicrob. Agents Chemother..

[6] Forsgren A., Schmeling D. (1977). Effect of antibiotics of chemotaxis of human leukocytes. Antimicrob. Agents Chemother..

[7] Hoeben D., Dosogne H., Heyneman R., Burvenich C. (1997). Effect of antibiotics on the phagocytotic and respiratory burst activity of bovine granulocytes. Eur. J. Pharmacol..

[8] Lai P.C., Schibler M.R., Walters J.D. (2015). Azithromycin enhances phagocytic killing of Aggregatibacter actinomycetemcomitans Y4 by human neutrophils. J. Periodontol..

[9] Jerjomiceva N., Seri H., Vollger L., Wang Y., Zeitouni N., Naim H.Y., von Kockritz-Blickwede M. (2014). Enrofloxacin enhances the formation of neutrophil extracellular traps in bovine granulocytes. J. Innate Immun..

[10] Schilcher K., Andreoni F., Uchiyama S., Ogawa T., Schuepbach R.A., Zinkernagel A.S. (2014). Increased neutrophil extracellular trap-mediated Staphylococcus aureus clearance through inhibition of nuclease activity by clindamycin and immunoglobulin. J. Infect. Dis..

[11] Konstantinidis T., Kambas K., Mitsios A., Panopoulou M., Tsironidou V., Dellaporta E., Kouklakis G., Arampatzioglou A., Angelidou I., Mitroulis I. (2016). Immunomodulatory Role of Clarithromycin in Acinetobacter Baumannii Infection via Formation of Neutrophil Extracellular Traps. Antimicrob. Agents Chemother..

[12] Hand W.L., Hand D.L. (2001). Characteristics and mechanisms of azithromycin accumulation and efflux in human polymorphonuclear leukocytes. Int. J. Antimicrob. Agents.

[13] Retsema J., Girard A., Schelkly W., Manousos M., Anderson M., Bright G., Borovoy R., Brennan L., Mason R. (1987). Spectrum and mode of action of azithromycin (CP-62,993), a new 15-membered-ring macrolide with improved potency against gram-negative organisms. Antimicrob. Agents Chemother..

[14] Bosnar M., Kelneric Z., Munic V., Erakovic V., Parnham M.J. (2005). Cellular uptake and efflux of azithromycin, erythromycin, clarithromycin, telithromycin, and cethromycin. Antimicrob. Agents Chemother..

[15] Keshari R.S., Verma A., Barthwal M.K., Dikshit M. (2013). Reactive oxygen species-induced activation of ERK and p38 MAPK mediates PMA-induced NETs release from human neutrophils. J. Cell. Biochem..

[16] Sood S. (2016). Chloramphenicol—A Potent Armament Against Multi-Drug Resistant (MDR) Gram Negative Bacilli?. J. Clin. Diagn. Res. JCDR.

[17] Yipp B.G., Kubes P. (2013). NETosis: How vital is it?. Blood.

[18] Van Vlem B., Vanholder R., De Paepe P., Vogelaers D., Ringoir S. (1996). Immunomodulating effects of antibiotics: Literature review. Infection.

[19] Hauser W.E., Remington J.S. (1982). Effect of antibiotics on the immune response. Am. J. Med..

[20] Kovaleva A., Remmelts H.H., Rijkers G.T., Hoepelman A.I., Biesma D.H., Oosterheert J.J. (2012). Immunomodulatory effects of macrolides during community-acquired pneumonia: A literature review. J. Antimicrob. Chemother..

[21] Zarogoulidis P., Papanas N., Kioumis I., Chatzaki E., Maltezos E., Zarogoulidis K. (2012). Macrolides: From in vitro anti-inflammatory and immunomodulatory properties to clinical practice in respiratory diseases. Eur. J. Clin. Pharmacol..

[22] Manda-Handzlik A., Bystrzycka W., Sieczkowska S., Demkow U., Ciepiela O. (2016). Antibiotics Modulate the Ability of Neutrophils to Release Neutrophil Extracellular Traps. Adv. Exp. Med. Biol..

[23] Bystrzycka W., Moskalik A., Sieczkowska S., Manda-Handzlik A., Demkow U., Ciepiela O. (2016). The effect of clindamycin and amoxicillin on neutrophil extracellular trap (NET) release. Cent.-Eur. J. Immunol..

[24] Konig M.F., Andrade F. (2016). A Critical Reappraisal of Neutrophil Extracellular Traps and NETosis Mimics Based on Differential Requirements for Protein Citrullination. Front. Immunol..

[25] Skrzeczynska-Moncznik J., Wlodarczyk A., Zabieglo K., Kapinska-Mrowiecka M., Marewicz E., Dubin A., Potempa J., Cichy J. (2012). Secretory leukocyte proteinase inhibitor-competent DNA deposits are potent stimulators of plasmacytoid dendritic cells: Implication for psoriasis. J. Immunol..

[26] Maueroder C., Mahajan A., Paulus S., Gosswein S., Hahn J., Kienhofer D., Biermann M.H., Tripal P., Friedrich R.P., Munoz L.E. (2016). Menage-a-Trois: The Ratio of Bicarbonate to CO2 and the pH Regulate the Capacity of Neutrophils to Form NETs. Front. Immunol..

[27] Koup J.R., Lau A.H., Brodsky B., Slaughter R.L. (1979). Chloramphenicol pharmacokinetics in hospitalized patients. Antimicrob. Agents Chemother..

[28] Matzneller P., Krasniqi S., Kinzig M., Sorgel F., Huttner S., Lackner E., Muller M., Zeitlinger M. (2013). Blood, tissue, and intracellular concentrations of azithromycin during and after end of therapy. Antimicrob. Agents Chemother..

